# Network meta-analysis of sacubitril/valsartan for the treatment of essential hypertension

**DOI:** 10.1007/s00392-022-02120-0

**Published:** 2022-11-03

**Authors:** Yaling Zhang, Xiaoyu Zhao, Hao Huang, Ming Li

**Affiliations:** 1 Department of Nephrology, Sichuan Provincial People’s Hospital, University of Electronic Science and Technology of China, Chengdu, 610072 China; 2 Department of Cardiovascular, Sichuan Provincial People’s Hospital, University of Electronic Science and Technology of China, Chengdu, 610072 China

**Keywords:** Hypertension, Systolic blood pressure, Diastolic blood pressure, Sacubitril/valsartan, Network meta-analysis

## Abstract

**Aim:**

Sacubitril/valsartan has been demonstrated to reduce blood pressure in hypertensive patients, but the best dose remains unclear. We performed this network meta-analysis to determine the comparative efficacy and safety of three available doses of sacubitril/valsartan (i.e., 100, 200, and 400 mg).

**Methods and results:**

We searched four databases for relevant studies published before January 2022. Mean systolic and diastolic blood pressures in the sitting position (msSBP and msDBP) and ambulatory condition (24-h maSBP and maDBP) and adverse events (AEs) were assessed. Nine randomized controlled trials (RCTs) involving 5474 patients were included. Sacubitril/valsartan 200 mg once daily was slightly better than 400 mg once daily in lowering 24-h maDBP (MD, 1.31 mmHg; 95% CI 0.61–2.01 mmHg), slightly better than 100 mg once daily in lowering 24-h maSBP (MD, − 3.70 mmHg; 95% CI  − 6.22 to − 1.18 mmHg) and 24-h maDBP (MD, − 2.98; 95% CI − 5.11 to − 0.85), and slightly better than Valsartan 160 mg once daily in lowering 24-h maSBP (MD, − 3.23 mmHg; 95% CI, − 5.25 to − 1.21). 400 mg once daily of sacubitril/valsartan was better than 200 mg once daily in lowering msDBP (MD, − 9.38 mmHg; 95% CI − 17.79 to − 0.97 mmHg). Interestingly, 400 mg once daily of sacubitril/valsartan had fewer trial-specified AEs than 200 mg once daily (OR, 0.74; 95%CI 0.55–0.99). There was no statistical difference for the remaining comparisons.

**Conclusions:**

In hypertensive patients, 200 mg once daily of sacubitril/valsartan may exert a greater reduction in ambulatory blood pressure than 100 mg once daily and 200 mg once daily may not be inferior to 400 mg once daily. Moreover, it is not clear that sacubitril/valsartan lowers blood pressure more than an angiotensin receptor blocker. Further trials are required to determine the incremental value of sacubitril/valsartan as an anti-hypertensive agent.

**Graphical abstract:**

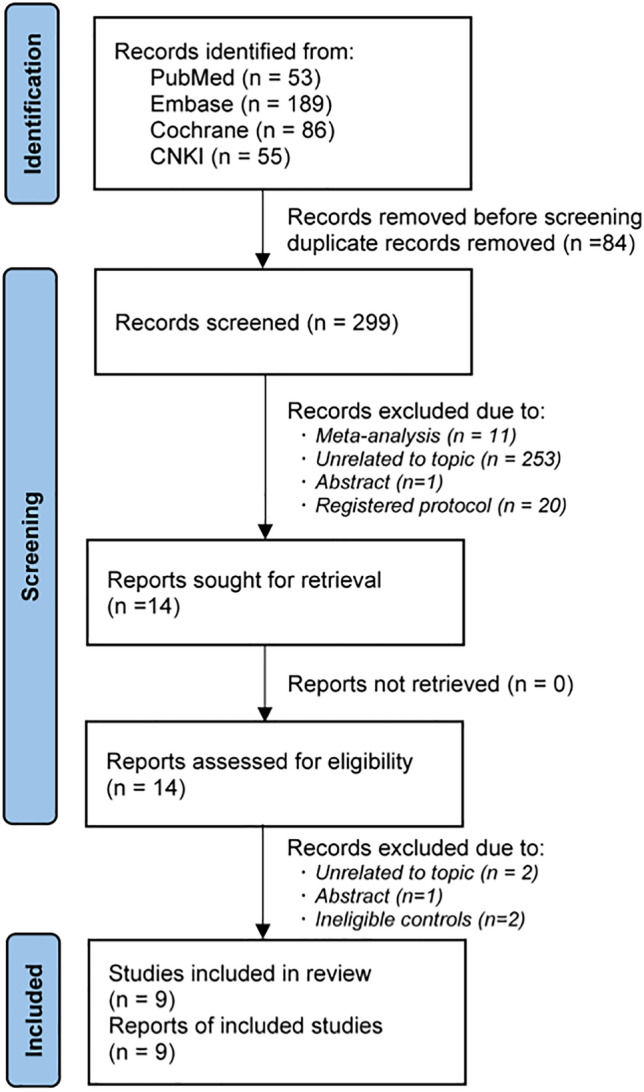

**Supplementary Information:**

The online version contains supplementary material available at 10.1007/s00392-022-02120-0.

## Introduction

Hypertension is one of the most common modifiable risk factors for cardiovascular diseases worldwide, such as stroke and congestive heart failure [1], and it is estimated that 1.5 billion populations will suffer from essential hypertension by 2025 [2]. Antihypertensive treatment has been proven to lower morbidity and mortality resulting from cardiovascular diseases [3], but the optimal treatment strategy for hypertension is still under investigation throughout the world [4, 5]. It is, therefore, essential to identify or even develop a novel agent for treating hypertensive patients.

Sacubitril/valsartan, also called LCZ696, is a first-in-class angiotensin receptor neprilysin inhibitor, which can provide a highly selective inhibition for neprilysin and the angiotensin receptors (ARBs) [6, 7]. It is noted that sacubitril/valsartan greatly exerts complementary therapeutic effects for the reduction of blood pressure by simultaneously inhibiting the neprilysin system and the angiotensin receptor. Studies revealed that sacubitril/valsartan could effectively reduce the ejection fraction and control blood pressure in patients with heart failure [8, 9]. Because of this, sacubitril/valsartan has been approved for treating heart failure with reduced ejection fraction.

Recently, researchers and practitioners have paid more attention to the potential value of sacubitril/valsartan in reducing blood pressure and performed several randomized controlled trials (RCTs) attempting to investigate its antihypertensive efficacy and safety among patients with essential hypertension [10–19]. In addition, several pairwise meta-analyses evaluating the efficacy and safety of sacubitril/valsartan in the treatment of hypertensive patients have also been reported, and all consistently suggested a higher reduction in blood pressure, although there was no significant difference in the risk of adverse events (AEs) in patients treated by sacubitril/valsartan compared with traditional therapies, such as angiotensin receptor blocker [20–24].

It is noted that multiple administration doses were available for sacubitril/valsartan in clinical practice, including 100 mg once daily, 200 mg once daily, and 400 mg once daily, which significantly confounded the clinical decision-making, because no meta-analysis has determined the relative efficacy and safety of these available doses of sacubitril/valsartan. Therefore, to provide a conclusive recommendation for clinicians to prescribe sacubitril/valsartan for hypertensive patients, we systematically evaluated the comparative efficacy and safety of three available doses of sacubitril/valsartan through combining available data using network meta-analysis technique [25].

## Methods

### Design of this study

We developed this network meta-analysis according to the Cochrane Handbook for reviewers [26]. We reported it following the Preferred Reporting Items for Systematic Reviews and Meta-Analysis (PRISMA) extension statement for reporting systematic reviews with network meta-analyses of health care interventions [27]. Unfortunately, we did not register the formal protocol on any platforms. This study did not require ethics approval and informed consent, because it was a network meta-analysis of published studies.

### Literature retrieval

Two independent reviewers performed a systematic literature retrieval in PubMed, Embase, the Cochrane Library, and China National Knowledge Infrastructure (CNKI) from the establishment data to January 2022. We developed the search strategy using the following keywords with a combination of medical subject headings and full-text words, including “hypertension,” “high blood pressure,” “sacubitril/valsartan,” “LCZ696”, and “random.” Language and publication status were not restricted for the literature search. Reference lists of included studies and reviews focused on a similar topic were also manually checked to identify additional studies. The detailed search strategy is summarized in Table S1. A third senior reviewer balanced any disagreement between two reviewers.

### Selection criteria

We developed selection criteria for this study according to previous meta-analyses [23, 24, 28]. Specifically speaking, studies were included if they met the following criteria: (1) patients who were enrolled in phase 3 randomized controlled trials (RCTs) were diagnosed with essential hypertension; (2) studies compared various doses (i.e., 100, 200, and 400 mg) of sacubitril/valsartan with each other or traditional therapies including Olmesartan (i.e., 20 and 40 mg) and Valsartan (i.e., 80, 160, and 320 mg); (3) studies reported the following outcomes, including mean systolic and diastolic blood pressure (msSBP and msDBP) in the sitting position, 24-h mean ambulatory systolic and diastolic blood pressure (maSBP and maDBP), and trial-specified adverse events (AEs); and (4) full-text was available for access. Moreover, we also excluded studies if they (1) were duplicate reports, (2) used ineligible design (e.g., observational studies and commentary), (3) designed ineligible controls (e.g., placebo or amlodipine), and (4) were conference abstract without sufficient data.

### Study selection

Two independent reviewers conducted the process of study selection. Specifically speaking, after removing duplicate records, two independent reviewers initially evaluated the eligibility of all unique records by screening the titles and abstracts. Finally, eligible studies were determined based on the evaluation of the full-texts. A third senior reviewer balanced any disagreement between the two reviewers.

### Data extraction

Two independent reviewers extracted the essential information from each eligible study utilizing the prepared data extraction sheet. Specifically speaking, the following data were extracted entirely: the name of the first author, publication year, the severity of hypertension, detailed information on the treatment plan, the number of patients randomized into each group, mean age of patients, basic body mass index (BMI), baseline blood pressure, duration, outcome data, and detailed information on the methodological quality. We would contact the corresponding author to add essential data if necessary. A third senior reviewer balanced any disagreement between the two reviewers.

### Geometry of the network plot

We used a network plot to depict the evidence structure of each outcome. A circle represents a treatment plan in the network plot, and its size was proportionate to the number of accumulated patients. Meanwhile, a solid line represents a direct comparison between two treatment plans and its width was proportionate to the number of direct comparisons [29].The same studies were included in this network meta-analysis for mean blood pressure in the sitting position and mean ambulatory blood pressure. Therefore, the same network plot was created for msSBP and msDBP as well as another individual network plot was for 24-h maSBP and 24-h maDBP.

### Risk of bias assessment

The methodological quality of each eligible study was evaluated by two independent reviewers using the Cochrane risk of bias assessment tool [30] from seven items, including random sequence generation, allocation concealment, blinding of personnel and participants, blinding of outcome assessment, incomplete outcome data, selective reporting, and other risk sources. Specifically speaking, each item was assessed as “high,” “unclear,” or “low” in risk bias according to the fact whether they were adequately performed. The overall level in methodological quality was rated as “high” if all seven items were labeled with “low” risk, as “low” if at least one of all items was labeled with “high” risk, or as “moderate” if at least one of all items was labeled with “unclear” risk but no item was “high” risk.

### Statistical analysis

We used mean difference (MD) with a corresponding 95% confidence interval (CI) to express the pooled estimates of all continuous variables, including msSBP, msDBP, 24-h maSBP, and 24-h maDBP. For trial-specified AEs, the pooled estimate was expressed using the odds ratio (OR) with 95% CI, because it is a categorical variable. We performed frequent network meta-analysis [31] to calculate all estimates on the basis of a random-effects model, because no completely identical studies were available in the real world [32]. The transitivity of across studies was a precondition of performing a network meta-analysis, we, therefore, first assessed it based on critical evaluation on clinical and methodological information [33, 34]. Then, we performed global [35] and local [36] consistency tests to determine which model should be selected for data synthesis. Moreover, we utilized the node-splitting method to assess the loop inconsistency [37, 38]. We also calculated the surface under the cumulative ranking curve (SUCRA) probabilities to rank the targeted doses of sacubitril/valsartan for each outcome [39]. Finally, we created comparison-adjusted funnel plots to evaluate publication bias [40]. Network meta-analysis was performed using STATA 14.0 (StataCorp LP, College Station, Texas, USA) with “network” command [41].

## Results

### Literature selection

**Fig. 1:**
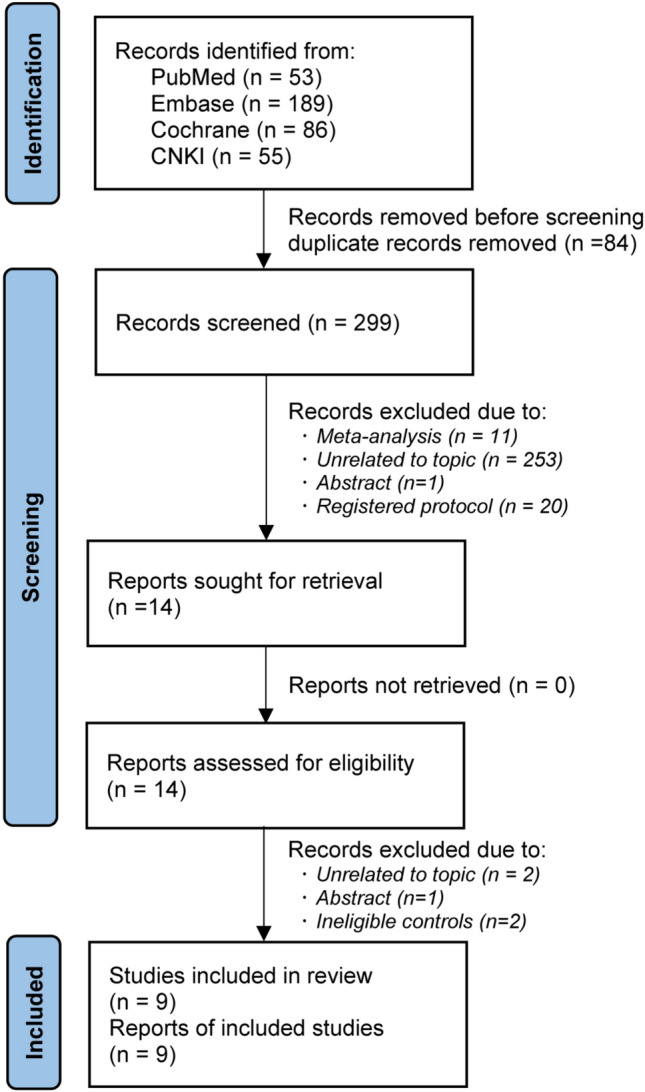
PRISMA flow chart of study selection. (*n*, number of articles)

A total of 383 records were identified from the initial search, but 84 duplicate records were marked and then removed by software. After an initial evaluation, 14 studies were retained for further evaluation based on full texts. Finally, 9 RCTs [10, 13–19, 42] were included for the final analysis, because five studies were excluded due to unrelated to a specific topic (*n* = 2), conference abstract without sufficient data (*n* = 1), and ineligible controls (*n* = 2). We created Fig. 1 to depict the procedural steps of study selection.

### Study characteristics

**Fig. 2:**
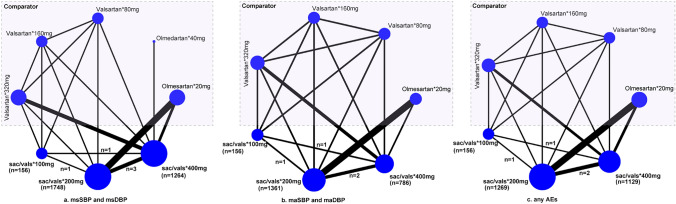
Network plot of all outcomes. The size of a circle is proportionate to the accumulated number of patients and the width of a solid line between two circles is proportional to the number of direct comparisons. AEs, adverse events

The baseline information of 9 included studies is summarized in Table 1. All studies were published between 2010 and 2022. The sample size of individual study ranged from 72 to 1435, with an accumulated number of 5474. Among 9 eligible studies, three studies were multiple-armed experiments and the others were two-armed experiments. Three administration doses were identified for sacubitril/valsartan in nine eligible studies, including 100, 200, and 400 mg admitted daily. All studies [10, 13–19, 42] reported mean blood pressure in sitting position, seven studies [10, 13, 16–19] reported mean ambulatory blood presume, and another eight [10, 15–19, 42] studies reported trial-specified AEs. The outcome data of all eligible studies are shown in Table 2. Network plots for mean blood pressure in the sitting position (i.e., 156, 1748, and 1264 patients were assigned to 100 m once daily, 200 mg once daily, and 400 mg once daily, respectively), mean ambulatory blood pressure (i.e., 156, 1361, and 786 patients were assigned to 100 once daily, 200 mg once daily, and 400 mg once daily, respectively), and trial-specified AEs (i.e., 156, 1269, and 1129 patients were assigned to 100 mg once daily, 200 mg once daily, 400 mg once daily, respectively) are shown in Fig. 2.

**Table 1:** Basic characteristics of the included studies (*n* = 9)

References	Race	Conditions	Regimes	Sample size	Age, years	BMI, kg/m^2^	SBP, mmHg	DBP, mmHg	Duration
Williams et al. [16]	not restricted	mild-to-moderate essential hypertension	Sac/vals 200 mg/day	229	68.2 ± 5.7	28.60 ± 4.47	160.4 ± 12.3	85.8 ± 8.6	52 weeks
			Olmesartan 20 mg/day	225	67.2 ± 6.0	29.10 ± 4.90	160.8 ± 15.6	85.8 ± 8.6	
Izzo et al. [13]	not restricted	mild-to-moderate essential hypertension	Sac/vals 400 mg/day	142	61.2 ± 10.6	29.30 ± 5.50	159.6 ± 7.0	90.9 ± 8.9	8 weeks
			Valsartan 320 mg/day	143	62.0 ± 11.5	30.00 ± 5.30	160.0 ± 7.3	90.2 ± 9.4	
Wang et al. [12]	Asians	salt-sensitive hypertension	Sac/vals 400 mg/day	36	55.7 ± 12.5	26.40 ± 3.80	147.0 ± 9.7	90.2 ± 6.9	4 weeks
			Valsartan 320mgday	36	58.9 ± 7.5	25.70 ± 2.90	147.5 ± 12.1	90.4 ± 7.2	
Supasyndh et al. [19]	Asians	elderly Asian	Sac/vals 200 mg/day	296	70.5 ± 4.7	24.30 ± 3.15	160.5 ± 8.4	84.6 ± 9.7	14 weeks
			Olmesartan 20 mg/day	292	70.9 ± 4.7	24.60 ± 3.24	160.0 ± 8.0	85.2 ± 9.8	
Schmieder et al. [14]	not restricted	stage 1 and 2 essential hypertensions	Sac/vals 400 mg/day	57	60.5 ± 7.8	28.10 ± 4.50	155.3 ± 9.0	92.7 ± 8.8	52 weeks
			Olmesartan 40 mg/day	57	59.2 ± 13.1	28.60 ± 3.90	155.0 ± 9.1	91.7 ± 8.7	
Huo et al. [17]	Asians	mild-to-moderate essential hypertension	Sac/vals 200 mg/day	479	57.5 ± 10.2	26.40 ± 3.91	158.0 ± 7.2	90.7 ± 9.4	8 weeks
			Sac/vals 400 mg/day	472	58.0 ± 9.7	26.30 ± 3.56	157.9 ± 6.7	89.8 ± 9.5	
			Olmesartan 20 mg/day	484	57.4 ± 10.1	26.40 ± 3.92	158.0 ± 6.5	90.8 ± 9.6	
Cheung et al. [18]	not restricted	mild-to-moderate essential hypertension	Sac/vals 200 mg/day	188	57.1 ± 10.2	30.50 ± 5.86	157.1 ± 9.5	90.4 ± 10.2	8 weeks
			Olmesartan 20 mg/day	187	58.0 ± 9.1	30.60 ± 5.09	157.8 ± 10.2	91.2 ± 8.9	
Ruilope et al. [10]	not restricted	mild-to-moderate essential hypertension	Sac/vals 100 mg/day	156	53.0 ± 10.4	n.a	154.9 ± 11.9	99.9 ± 3.6	8 weeks
			Sac/vals 200 mg/day	169	54.0 ± 9.7	n.a	156.8 ± 12.0	99.9 ± 4.1	
			Sac/vals 400 mg/day	172	52.0 ± 10.9	n.a	156.3 ± 12.3	100.4 ± 4.1	
			Valsartan 80 mg/day	163	53.0 ± 9.6	n.a	154.8 ± 10.5	99.5 ± 4.1	
			Valsartan 160 mg/day	166	53.0 ± 9.7	n.a	155.3 ± 10.8	99.8 ± 4.4	
			Valsartan 320 mg/day	164	53.0 ± 10.1	n.a	156.0 ± 11.5	99.5 ± 3.6	
Rakugi et al. [42]	Asians	mild-to-moderate essential hypertension	Sac/vals 200 mg/day	387	57.9 ± 10.9	25.4 ± 3.7	157.7 ± 6.9	94.3 ± 9.4	8 weeks
			Sac/vals 400 mg/day	385	58.7 ± 10.5	25.3 ± 3.9	158.4 ± 7.3	94.8 ± 9.8	
			Olmesartan 20 mg/day	389	59.6 ± 10.5	25.6 ± 3.8	157.6 ± 6.8	93.8 ± 9.7	

*Sac/vals* sacubitril-valsartan, *BMI* body mass index, *SBP* systolic blood pressure, *DBP* diastolic blood pressure

**Table 2:** Outcomes of included studies (*n* = 9)

References	Regimes	msSBP	msDBP	maSBP	maDBP	AEs
Williams et al. [16]	Sac/Val 200 mg/day	− 13.7 ± 16.2	− 5.9 ± 8.8	− 13.3 ± 7.8	− 7.4 ± 4.7	132
	Olmesartan 20 mg/day	− 9.9 ± 16.5	− 4.9 ± 8.8	− 9.1 ± 7.9	− 5.5 ± 4.6	121
Izzo et al. [13]	Sac/Val 400 mg/day	− 21.9 ± 22.6	− 9.7 ± 15.5	13.0 ± 6.7	− 6.2 ± 4.2	n.a
	Valsartan 320 mg/day	− 16.3 ± 22.2	− 7.2 ± 13.2	− 9.6 ± 6.9	− 5.2 ± 4.2	n.a
Wang et al. [12]	Sac/Val 400 mg/day	− 13.3 ± 13.2	− 6.2 ± 8.4	n.a	n.a	12
	Valsartan 320 mg/day	− 5.8 ± 14.0	− 4.2 ± 9.3	n.a	n.a	12
Supasyndh et al. [19]	Sac/Val 200 mg/day	− 22.7 ± 15.6	− 8.6 ± 8.1	− 14.2 ± 7.00	− 7.00 ± 3.9	141
	Olmesartan 20 mg/day	− 16.1 ± 15.7	− 6.5 ± 8.2	− 9.1 ± 7.0	− 4.5 ± 3.9	113
Schmieder et al. [14]	Sac/Val 400 mg/day	− 25.4 ± 12.5	− 11.6 ± 8.8	n.a	n.a	n.a
	Olmesartan 40 mg/day	− 22.8 ± 14.2	− 11.5 ± 8.9	n.a	n.a	n.a
Huo et al. [17]	Sac/Val 200 mg/day	− 20.5 ± 13.3	− 8.1 ± 8.1	− 12.1 ± 6.7	− 6.4 ± 4.3	143
	Sac/Val 400 mg/day	21.7 ± 13.4	− 8.8 ± 8.2	− 12.8 ± 6.7	− 6.5 ± 4.2	132
	Olmesartan 20 mg/day	− 18.2 ± 13.4	− 6.9 ± 8.1	− 10.3 ± 2.6	− 5.6 ± 4.2	134
Cheung et al. [18]	Sac/Val 200 mg/day	− 14.2 ± 17.6	− 7.5 ± 9.6	− 4.3 ± 7.8	− 2.3 ± 5.0	44
	Olmesartan 20 mg/day	− 10.0 ± 17.6	− 4.5 ± 9.7	− 1.1 ± 7.7	− 0.4 ± 5.00	41
Ruilope et al. [10]	Sac/Val 100 mg/day	− 6.0 ± 13.8	− 3.2 ± 8.8	− 7.8 ± 11.8	− 3.8 ± 12.9	36
	Sac/Val 200 mg/day	− 18.7 ± 14.3	12.9 ± 9.1	− 11.5 ± 7.3	− 6.8 ± 4.5	40
	Sac/Val 400 mg/day	− 20.2 ± 14.3	− 13.6 ± 9.1	14.6 ± 7.5	− 8.3 ± 5.2	50
	Valsartan 80 mg/day	− 4.7 ± 13.8	− 2.4 ± 8.8	− 7.2 ± 11.8	− 4.8 ± 12.9	36
	Valsartan 160 mg/day	− 13.4 ± 14.3	− 10.0 ± 9.1	− 8.3 ± 7	− 6.3 ± 4.9	34
	Valsartan 320 mg/day	− 14.2 ± 14.3	− 10.9 ± 9.1	− 9.4 ± 7.1	− 7.1 ± 4.9	38
Rakugi et al. [42]	Sac/Val 200 mg/day	− 18.2 ± 12.5	− 9.5 ± 8.5	n.a	n.a	135
	Sac/Val 400 mg/day	− 20.2 ± 13.5	− 10.5 ± 9.7	n.a	n.a	136
	Olmesartan 20 mg/day	− 13.2 ± 15.3	− 6.9 ± 9.8	n.a	n.a	152

*msSBP* mean sitting systolic blood pressure, *msDBP* mean sitting diastolic blood pressure, *maSBP* mean ambulatory SBP, *maDBP* mean ambulatory DBP, *AEs* adverse events, *n.a.* not available

### Risk of bias

Among nine eligible studies, all studies [10, 13–19, 42] generated random sequence using appropriate methods and correctly concealed the allocation process. All studies [10, 13–19, 42] were rated as low risk in performance bias, but only three studies [10, 13, 17] clearly reported the methods of blinding outcome assessment. Moreover, three [10, 17, 42] and four [10, 13–15] studies were definitively identified to completely report outcome data and anticipated outcomes. Only one study [10] was definitively determined as low risk in other sources. The detailed risk of bias of all studies is shown in Fig. S1. Finally, the overall quality was appraised as a moderate level.

### Meta-analysis of msSBP and msDBP

All studies [10, 13–19, 42] reported data of msSBP, and we did not detect substantial inconsistency based on the global inconsistency test (Fig. S2) and the node-split test (Fig. S3). Therefore, the consistency model was selected to calculate the pooled estimate. Network meta-analysis suggested that there was no statistical difference of sacubitril/valsartan 100 mg once daily vs. Valsartan 80 mg once daily, sacubitril/valsartan 200 mg once daily vs. Olmesartan 20 mg once daily and Valsartan 160 mg once daily, and sacubitril/valsartan 400 mg once daily vs. Olmesartan 40 mg once daily and Valsartan 320 mg once daily (Fig. S4). In addition, there was no significant difference in the reduction of msSBP between three doses of sacubitril/valsartan (Fig. S4). The numerical results can be accessed in Table 3. However, results of SUCRA analysis revealed that sacubitril/valsartan 200 mg once daily had the highest probability of being best (87.1%), followed by Olmesartan 20 mg once daily (78.6%), sacubitril/valsartan 400 mg once daily (49.5%), Valsartan 160 mg once daily (47.3%), Valsartan 40 mg once daily (44.5%), Valsartan 320 mg once daily (39.5%), 100 mg once daily (28.3%), and Valsartan 80 mg once daily (25.1%) (Fig. S4).

**Table 3:** Relative results of different comparisons in terms of different outcomes

Comparison (continuous outcomes)	Number of patients	MD with 95%CI, mmHg
*msSBP*		
sac/vals*200 mg vs. sac/vals*100 mg	1748 vs. 156	− 21.46 (− 47.78 to 4.86)
sac/vals*400 mg vs. sac/vals*100 mg	1264 vs. 156	− 8.44 (− 33.84 to 16.96)
sac/vals*400 mg vs. sac/vals*200 mg	1264 vs. 1748	13.02 (− 2.55 to 28.59)
sac/vals*100 mg vs. Valsartan 80 mg	156 vs. 163	− 1.30 (− 30.71 to 28.11)
sac/vals*200 mg vs. Olmesartan 20 mg	1579 vs. 1577	− 2.63 (− 15.50 to 10.24)
sac/vals*200 mg vs. Valsartan 160 mg	169 vs. 166	− 14.06 (− 40.38 to 12.26)
sac/vals*400 mg vs. Olmesartan 40 mg	57 vs. 57	− 2.59 (− 32.25 to 27.06)
sac/vals*400 mg vs. Valsartan 320 mg	350 vs. 343	− 3.37 (− 19.92 to 13.17)
*msDBP*		
sac/vals*200 mg vs. sac/vals*100 mg	1748 vs. 156	5.93 (− 8.29 to 20.16)
sac/vals*400 mg vs. sac/vals*100 mg	1264 vs. 156	− 3.45 (− 17.18 to 10.27)
sac/vals*400 mg vs. sac/vals*200 mg	1264 vs. 1748	− 9.38 (− 17.79 to − 0.97)
sac/vals*100 mg vs. Valsartan 80 mg	156 vs. 163	− 0.83 (− 16.72 to 15.06)
sac/vals*200 mg vs. Olmesartan 20 mg	1579 vs. 1577	0.04 (− 6.90 to 6.98)
sac/vals*200 mg vs. Valsartan 160 mg	169 vs. 166	12.69 (− 1.53 to 26.92)
sac/vals*400 mg vs. Olmesartan 40 mg	57 vs. 57	− 0.10 (− 16.21 to 16.00)
sac/vals*400 mg vs. Valsartan 320 mg	350 vs. 343	1.10 (− 7.88 to 10.07)
*maSBP*		
sac/vals*200 mg vs. sac/vals*100 mg	1748 vs. 156	− 3.70 (− 6.22 to − 1.18)
sac/vals*400 mg vs. sac/vals*100 mg	1264 vs. 156	− 1.96 (− 5.08 to 1.16)
sac/vals*400 mg vs. sac/vals*200 mg	879 vs. 1361	1.74 (− 0.10 to 3.58)
sac/vals*100 mg vs. Valsartan 80 mg	156 vs. 163	− 0.60 (− 3.50 to 2.30)
sac/vals*200 mg vs. Olmesartan 20 mg	1192 vs. 1188	− 0.54 (− 3.29 to 2.22)
sac/vals*200 mg vs. Valsartan 160 mg	169 vs. 166	− 3.23 (− 5.25 to − 1.21)
sac/vals*400 mg vs. Olmesartan 40 mg	n.a	n.a
sac/vals*400 mg vs. Valsartan 320 mg	314 vs. 307	− 0.34 (− 3.08 to 2.40)
*maDBP*		
sac/vals*200 mg vs. sac/vals*100 mg	1748 vs. 156	− 2.98 (− 5.11 to − 0.85)
sac/vals*400 mg vs. sac/vals*100 mg	1264 vs. 156	− 1.67 (− 3.91 to 0.57)
sac/vals*400 mg vs. sac/vals*200 mg	879 vs. 1361	1.31 (0.61 to 2.01)
sac/vals*100 mg vs. Valsartan 80 mg	156 vs. 163	1.00 (− 1.83 to 3.83)
sac/vals*200 mg vs. Olmesartan 20 mg	1192 vs. 1188	0.76 (− 1.42 to 2.94)
sac/vals*200 mg vs. Valsartan 160 mg	169 vs. 166	− 0.53 (− 1.54 to 0.48)
sac/vals*400 mg vs. Olmesartan 40 mg	n.a	n.a
sac/vals*400 mg vs. Valsartan 320 mg	314 vs. 307	1.66 (0.43 to 2.89)

Comparison (dichotomous outcome)	Number of patients	OR with 95% CI
*Trial-specified AEs*		
sac/vals*200 mg vs. sac/vals*100 mg	1748 vs. 156	1.03 (0.62–1.73)
sac/vals*400 mg vs. sac/vals*100 mg	1264 vs. 156	0.76 (0.42–1.38)
sac/vals*400 mg vs. sac/vals*200 mg	1264 vs. 1748	0.74 (0.55–0.99)
sac/vals*100 mg vs. Valsartan 80 mg	156 vs. 163	1.06 (0.63–1.79)
sac/vals*200 mg vs. Olmesartan 20 mg	1579 vs. 1577	1.22 (0.70–2.14)
sac/vals*200 mg vs. Valsartan 160 mg	169 vs. 166	1.20 (0.72–2.02)
sac/vals*400 mg vs. Olmesartan 40 mg	n.a	n.a
sac/vals*400 mg vs. Valsartan 320 mg	208 vs. 200	0.76 (0.42–1.36)

*msSBP* mean sitting systolic blood pressure, *msDBP* mean sitting diastolic blood pressure, *maSBP* mean ambulatory SBP, *maDBP* mean ambulatory DBP, *MD* mean difference, *CI* confidence interval, *n.a.* not applicable

All studies [10, 13–19, 42] reported data of msDBP, but we detected substantial inconsistency based on the global inconsistency test (Fig. S2) and the node-split test (Fig. S3). Therefore, we selected the inconsistency model to calculate the pooled estimate. Network meta-analysis suggested that there was no statistical difference of sacubitril/valsartan 100 mg once daily vs. Valsartan 80 mg once daily, sacubitril/valsartan 200 mg once daily vs. Olmesartan 20 mg once daily and Valsartan 160 mg once daily, and sacubitril/valsartan 400 mg once daily vs. Olmesartan 40 mg once daily and Valsartan 320 mg once daily (Fig. S4). In addition, 400 mg once daily of sacubitril/valsartan was superior to 200 mg once daily in reducing msDBP (MD, − 9.38 mmHg; 95% CI, − 17.79 to − 0.97 mmHg), although the other two comparisons were not statistically significant (Fig. S4). The numerical results can be accessed in Table 3. Furthermore, results of SUCRA analysis revealed that Valsartan 160 mg once daily had the highest probability of being best (77.1%), followed by Valsartan 320 mg once daily (71.2%), 400 mg once daily of sacubitril/valsartan (66.2%), 100 mg once daily of sacubitril/valsartan (47.2%), Valsartan 80 mg once daily (42.3%), Olmesartan 20 mg once daily (18.0%), and 200 mg once daily of sacubitril/valsartan (16.8%) (Fig. S4).

### Meta-analysis of 24-h maSBP and 24-h maDBP

Seven studies [10, 13, 16–19] reported data of 24-h maSBP, but we detected substantial inconsistency based on the global inconsistency test (Fig. S2) and the node-split test (Fig. S3). We, therefore, used an inconsistency model to calculate the pooled estimate. Network meta-analysis suggested that there was no statistical difference of 100 mg once daily of sacubitril/valsartan vs. Valsartan 80 mg once daily, 200 mg once daily of sacubitril/valsartan vs. Olmesartan 20 mg once daily, and 400 mg once daily of sacubitril/valsartan vs. Valsartan 320 mg once daily; however, 200 mg once daily of sacubitril/valsartan was inferior to Valsartan 160 mg once daily (Fig. S5). In addition, 200 mg once daily of sacubitril/valsartan was superior to 100 mg once daily in reducing 24-h maSBP (MD, -3.70 mmHg; 95% CI − 6.22 to − 1.18 mmHg), although the other two comparisons were not statistically significant (Fig. S5). The numerical results can be accessed in Table 3. Furthermore, results of SUCRA analysis revealed that 200 mg once daily of sacubitril/valsartan had the highest probability of being best (99.0%), followed by 400 mg once daily of sacubitril/valsartan (72.2%), Valsartan 320 mg once daily (68.3%), Valsartan 160 mg once daily (42.3%), 100 mg once daily of sacubitril/valsartan (31.3%), Olmesartan 20 mg once daily (18.6%), and Valsartan 80 mg once daily (18.3%) (Fig. S5).

These same studies [10, 13, 16–19] also reported data of 24-h maDBP and substantial inconsistency was detected by the global (Fig. S2) and local (Fig. S3) inconsistency tests. The inconsistency model was, therefore, selected to calculate the pooled estimate. Network meta-analysis suggested that there was no statistical difference of 100 mg once daily of sacubitril/valsartan vs. Valsartan 80 mg once daily, 200 mg once daily of sacubitril/valsartan vs. Olmesartan 20 mg once daily and Valsartan 160 mg once daily; however, 400 mg once daily of sacubitril/valsartan was better than Valsartan 320 mg once daily (Fig. S5). In addition, 200 mg once daily of sacubitril/valsartan was superior to 100 mg once daily (MD, − 2.98; 95% CI − 5.11 to − 0.85) and 400 mg once daily in reducing 24-h maDBP (MD, 1.31 mmHg; 95% CI 0.61–2.01 mmHg) (Fig. S5). The numerical results can be accessed in Table 3. Results of SUCRA analysis revealed that Valsartan 320 mg once daily had the highest probability of being best (94.5%), followed by 200 mg once daily of sacubitril/valsartan (84.4%), Valsartan 160 mg once daily (66.6%), 400 mg once daily of sacubitril/valsartan (46.0%), Valsartan 80 mg once daily (29.7%), Olmesartan 20 mg once daily (19.2%), and 100 mg once daily of sacubitril/valsartan (9.7%) (Fig. S5).

### Meta-analysis of trial-specified AEs

A total of eight studies [10, 15–19, 42] reported data of trial-specified AEs, but no substantial inconsistency was detected based on the global inconsistency test (Fig. S2) and the node-split test (Fig. S3). We, therefore, used the consistency model to calculate the pooled estimate. Network meta-analysis suggested that there was no statistical difference of 100 mg once daily of sacubitril/valsartan vs. Valsartan 80 mg once daily, 200 mg once daily of sacubitril/valsartan vs. Olmesartan 20 mg once daily and Valsartan 160 mg once daily, and 400 mg once daily of sacubitril/valsartan vs. Valsartan 320 mg once daily (Fig. S6). In addition, 400 mg once daily of sacubitril/valsartan was associated with a lower incidence of trial-specified AEs than 200 mg once daily (OR, 0.74; 95% CI 0.55–0.99); however, no statistical difference was available for another two comparisons (Fig. S6). Results of SUCRA analysis revealed that 400 mg once daily of sacubitril/valsartan had the highest probability of being safest (79.6%), followed by 20 mg once daily of Olmesartan (66.9%), 160 mg once daily of Valsartan (60.2%), 80 mg once daily of Valsartan (45.6%), 100 mg once daily of sacubitril/valsartan (36.6%), 320 mg once daily of Valsartan (35.3%), and 200 mg once daily of sacubitril/valsartan (25.9%) (Fig. S6).

### Radar presentation

**Fig. 3:**
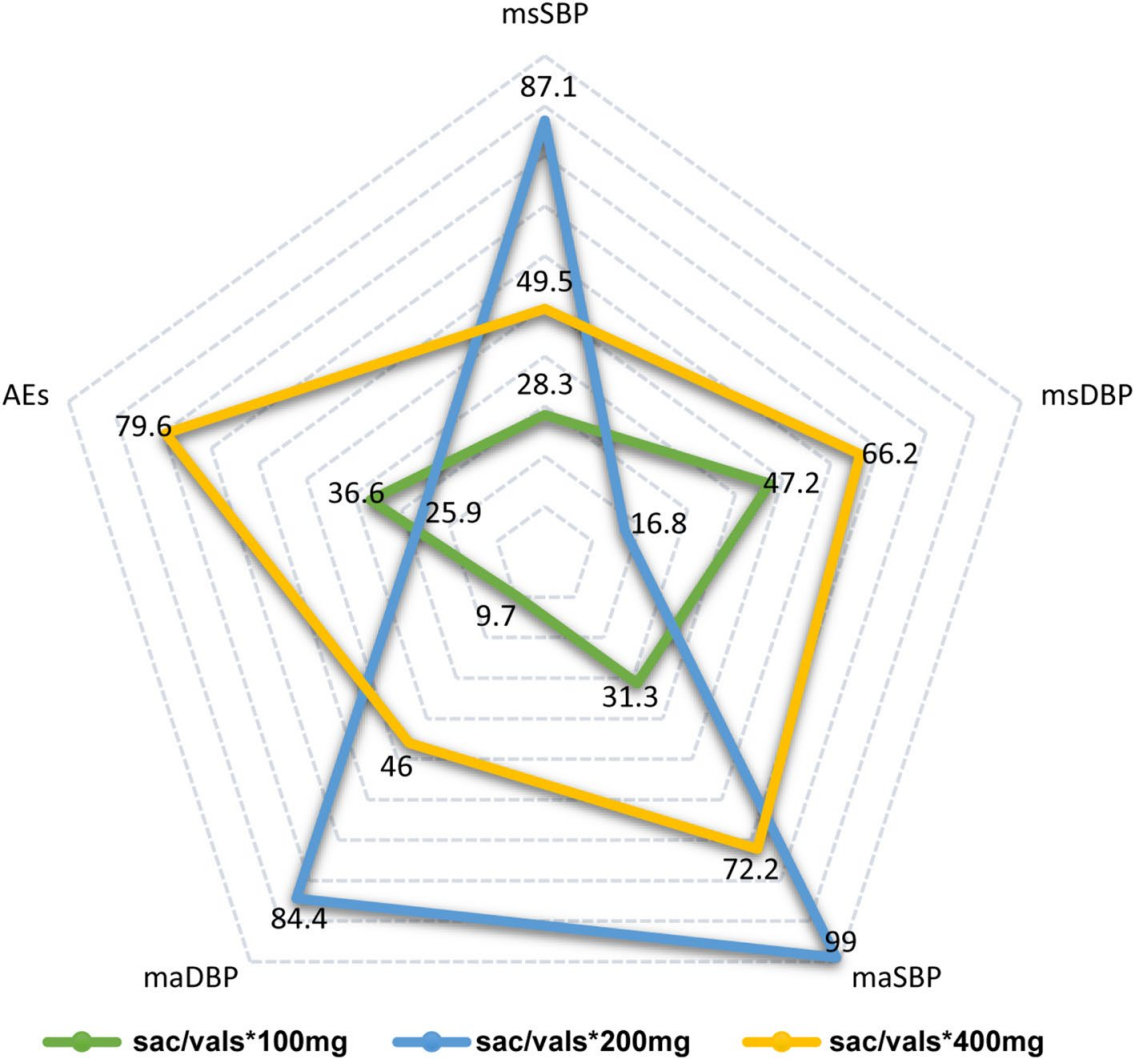
Radar plot of various doses for all outcomes. Five angles of this radar plot represent outcomes. Each pentagon in the radar plot refers to the ranking probability, and the larger pentagon indicated higher ranking probability

We used a radar plot to determine which treatment option may be optimal for a specific outcome, which was created based on the results of the SUCRA analysis. As displayed in Fig. 3, 200 mg once daily of sacubitril/valsartan located in the outer area of radar plot for msSBP, 24-h maSBP, and 24-h maDBP, indicating that it might be the best treatment option for lowering msSBP, 24-h maSBP, and 24-h maDBP. In terms of reducing msDBP, 400 mg once daily of sacubitril/valsartan located the outer area of the radar plot. Therefore, 400 mg once daily sacubitril/valsartan might be the best treatment option for msDBP. Moreover, 400 mg once daily sacubitril/valsartan might be the safest option, because it located in the outer area in the radar plot for AEs.

### Inconsistency and publication bias

As shown in Fig. S7, the node-split method did not determine loop-closed inconsistency for msSBP and trial-specified AEs, but loop-closed inconsistency was available for msDBP, 24-h maSBP, and 24-h maDBP. Comparison-adjusted funnel plot was created to evaluate publication bias. As shown in Fig. S8, asymmetric outlines were constructed for all outcomes. This indicated that pooled results might be negatively influenced by publication bias.

## Discussion

Several therapeutic strategies were available for essential hypertension; however, the management of hypertension remains suboptimal throughout the world [5]. This network meta-analysis mainly evaluated the comparative efficacy and safety of 100 mg once daily, 200 mg once daily, and 400 mg once daily of sacubitril/valsartan in the treatment of essential hypertension and found that 400 mg once daily of sacubitril/valsartan achieved more reduction in msDBP, although 200 mg once daily of sacubitril/valsartan had more reduction in 24-h maDBP. It is noted that 400 mg once daily of sacubitril/valsartan leaded to fewer trial-specified AEs than 200 mg once daily of sacubitril/valsartan. Moreover, no statistical differences in all outcomes were detected between different dosages of sacubitril/valsartan and corresponding dosages of ARBs.

As a novel cardiovascular medicine, sacubitril/valsartan combines the action angiotensin receptor blocker with a neprilysin inhibitor in a 1:1 ratio [43]. Therefore, it can effectively increase natriuretic peptides by simultaneously inhibiting the neprilysin system and renin–angiotensin–aldosterone system for increasing [44–47], and finally provide dual antihypertensive effects. Sacubitril/valsartan was, therefore, expected to have a more significant antihypertensive effect than ARBs alone [28]. Previous meta-analyses [20–24, 28] have demonstrated that the proportion of achieving target blood pressure among hypertensive patients treated by sacubitril/valsartan was significantly higher than that in patients receiving ARBs alone. Nevertheless, it remains unclear which dose of sacubitril/valsartan may be the optimal option due to the presence of various administration doses were prescribed clinically. Compare to previous meta-analyses, the present meta-analysis first comprehensively evaluated the comparative efficacy of three doses of sacubitril/valsartan for the treatment of hypertensive patients, and suggested that 400 mg once daily of sacubitril/valsartan might be the optimal strategy for blood pressure reduction.

Although the therapeutic efficacy of sacubitril/valsartan has been extensively emphasized and investigated in treating hypertensive patients, its safety in treating hypertension has recently received increasing attention. In this network meta-analysis, we found that, compared with 100 mg once daily or 200 mg once daily, 400 mg once daily of sacubitril/valsartan was associated with the lowest incidence of AEs. However, a systematic review and meta-analysis of investigating the safety and tolerability of sacubitril/valsartan concluded that there were no significant differences in the risk of angioedema, cough, hyperkalemia, and acute kidney injury with sacubitril/valsartan compared with any other active controls after combining results of 20 eligible studies [48]. Therefore, therapeutic efficacy in reducing blood pressure should be especially emphasized when one determines which dose of sacubitril/valsartan should be prescribed.

Nowadays, several pairwise meta-analyses are available to investigate the role of sacubitril/valsartan in treating hypertensive patients [21, 23, 24]. As the first study investigating the comparative efficacy and safety of sacubitril/valsartan, the present network meta-analysis has several strengths as follows: (1) we first imposed the network meta-analysis technique to determine the differences between various doses of sacubitril/valsartan in efficacy and safety, which provided a more definitive recommendation for decision-making; (2) only RCTs were included for data analysis, which greatly enhanced the reliability of pooled results; and (3) radar plot was used to determine and showed the subtle differences of various doses, which provided a reliable evidence base for making a recommendation.

However, this network meta-analysis has some limitations: (1) the limited number of eligible studies are unavoidable problems. More importantly, of the 9 eligible studies, one study only enrolled 72 patients and only 156 patients were assigned to receive sacubitril/valsartan 100 mg/day, which might introduce bias; (2) although all eligible studies were rated as low risk in selection bias and performance bias, most of them were judged as unclear risk in detection bias, reporting bias, attrition bias, and other bias sources. Obviously, unclear risk might introduce potential bias for pooled results; (3) substantial inconsistency was identified for msDBP, 24-h maSBP, and 24-h maDBP, we, therefore, used the inconsistency model to estimate the pooled results. However, we could not eliminate the negative impact of inconsistency on pooled results; (4) a comparison-adjusted funnel plot for all outcomes suggested the possible presence of publication bias, which also generated a negative influence on our findings; (5) a formal protocol of this network meta-analysis was not registered publicly; however, we developed it in strict accordance with recommendations made by the Cochrane handbook for reviewers. Meanwhile, we imposed a PRISMA–NMA checklist to facilitate the reporting of this network meta-analysis, which also substantially strengthened the reliability of pooled results; and (6) we did not simply rank all regimens according to the results of the SUCRA analysis. Specifically, a false conclusion may be generated only according to the SUCRA analysis, because the SUCRA analysis could not truly reveal the subtle difference between different dosages.

## Conclusions

This network meta-analysis suggests that 200 mg once daily of sacubitril/valsartan may exert a greater reduction in blood pressure than 100 mg once daily. Although 400monce daily did not appear to reduce blood pressure more than 200 mg once daily, 400 mg once daily appeared at least as well tolerated as lower doses. However, it is unclear whether any dose of sacubitril/valsartan lowers blood pressure more than a substantial dose of ARBs. The statistical power of this network meta-analysis for some comparisons was limited; further well-designed trials are required to investigate a potential role for sacubitril/valsartan in the treatment of hypertension.

## Supplementary Information

Below is the link to the electronic supplementary material.

Supplementary file1 (DOCX 10171 kb)

## Data Availability

The data underlying this article are available in the article and in its online supplementary material.
