# Supplementary material for: Network meta-analysis of sacubitril/valsartan for the treatment of essential hypertension
Source: Clin Res Cardiol. 2022 Nov 3;112(7):855–67. doi: 10.1007/s00392-022-02120-0 (PMC10293449; doi:10.1007/s00392-022-02120-0)
Supplement: Supplementary file 1 — Supplementary file1 (DOCX 10171 kb) [file 392_2022_2120_MOESM1_ESM.docx]

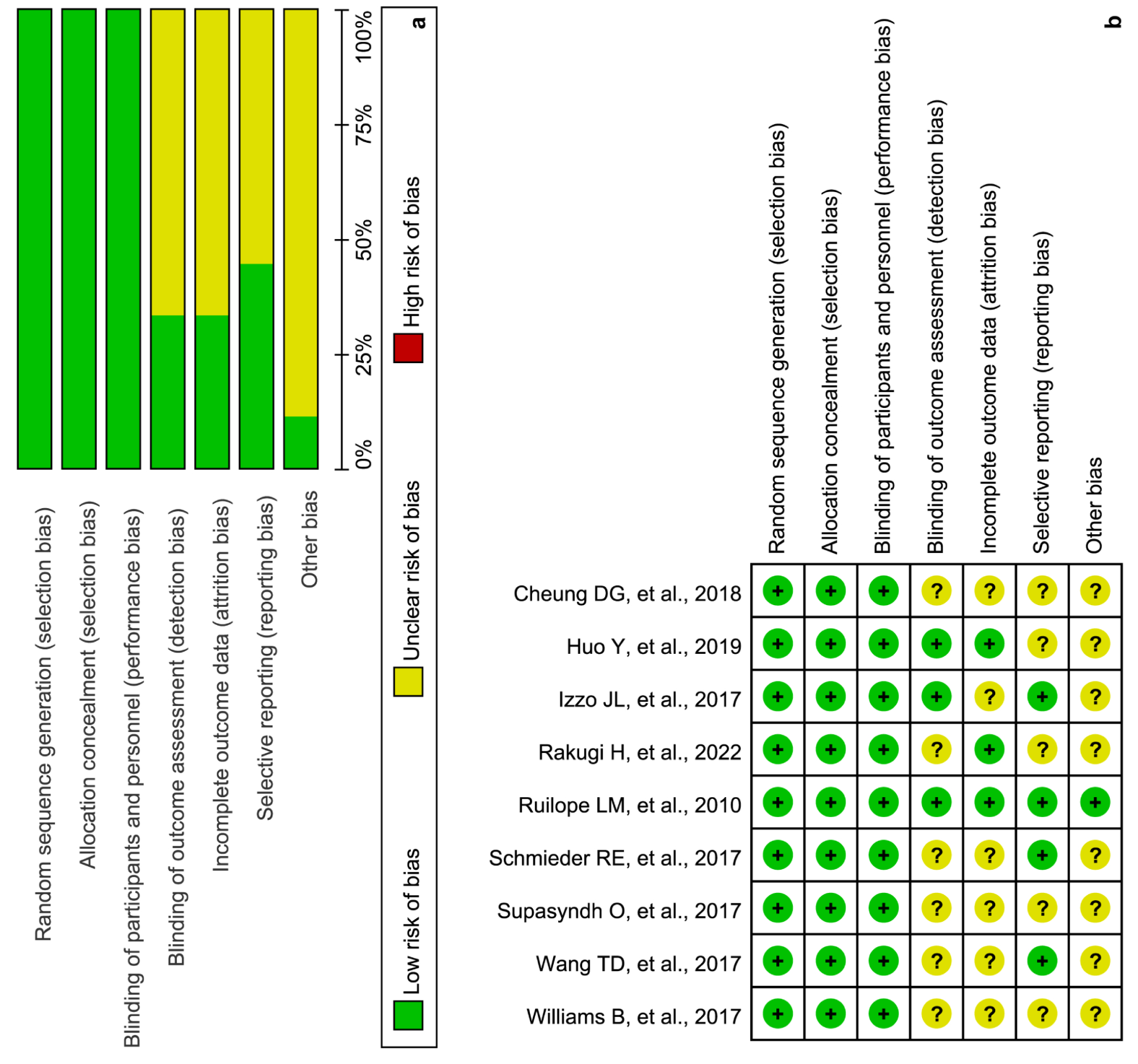


**Figure S1. Risk of bias.**


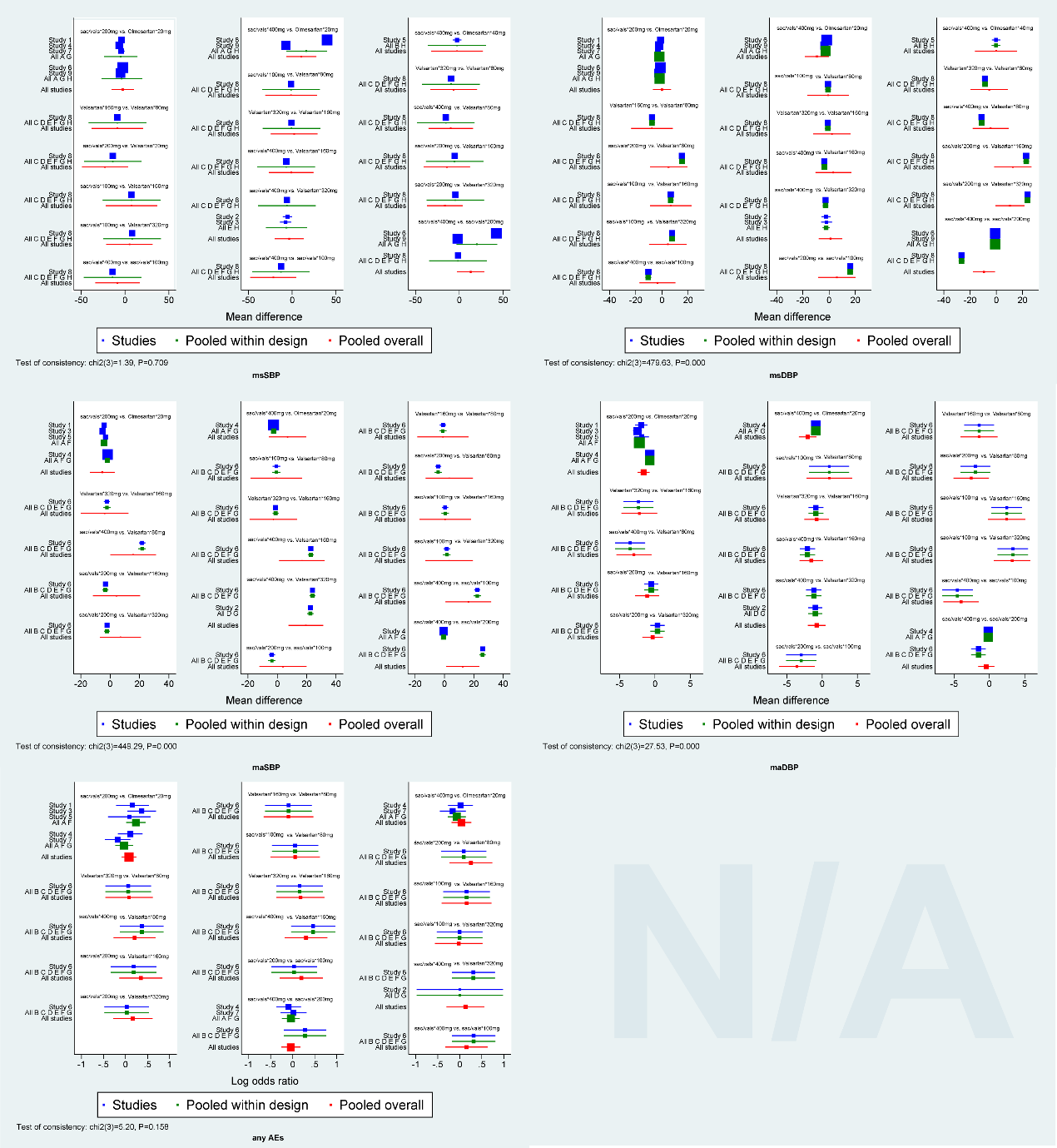


**Figure S2. Global consistency model tests for all outcomes.** Sac/vals, sacubitril/valsartan; msSBP, mean systolic blood pressure in the sitting position; msDBP, mean diastolic blood pressure in the sitting position; maSBP, mean ambulatory systolic blood pressure; maDBP, mean ambulatory diastolic blood pressure; AEs, adverse events.


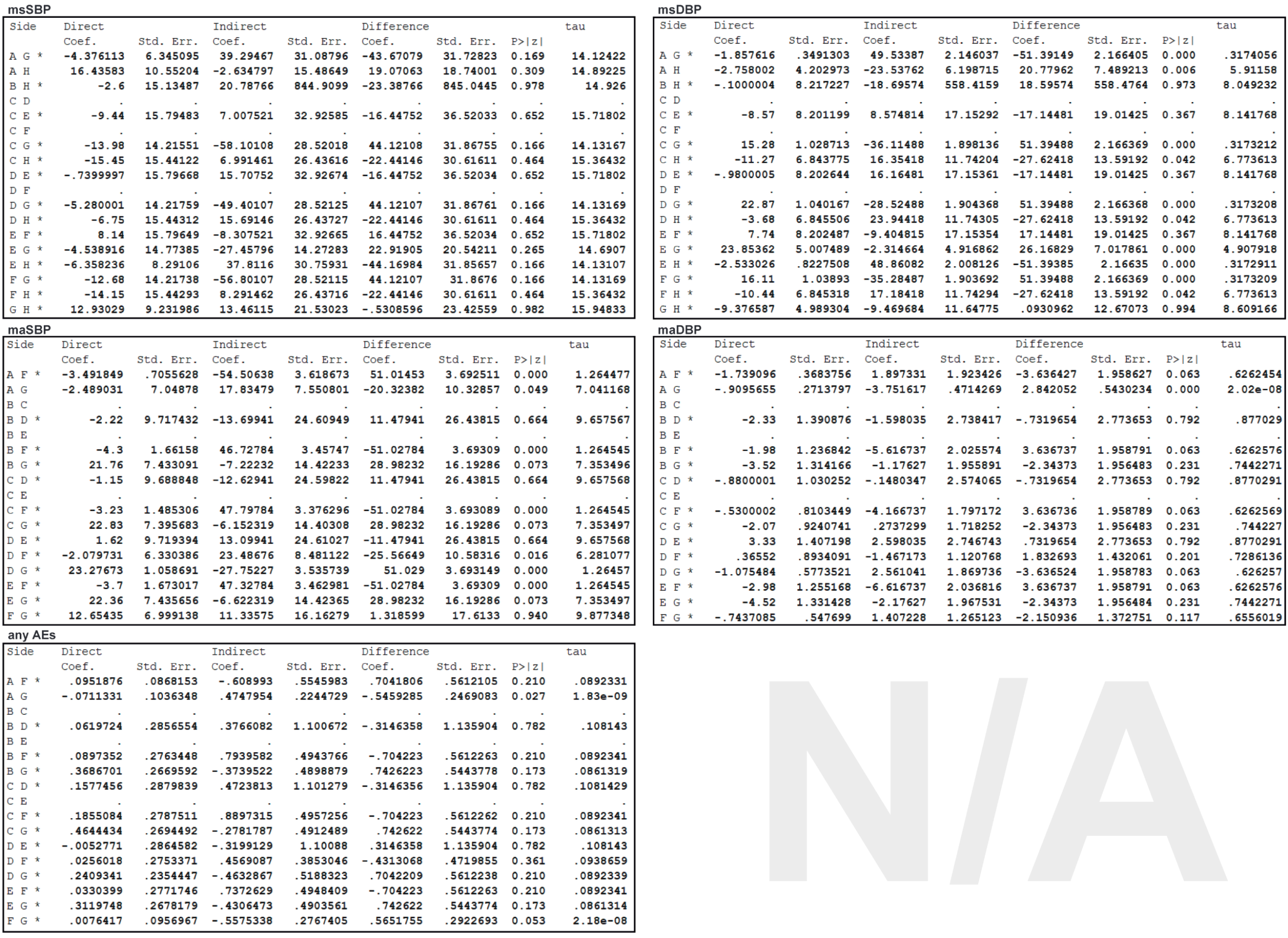

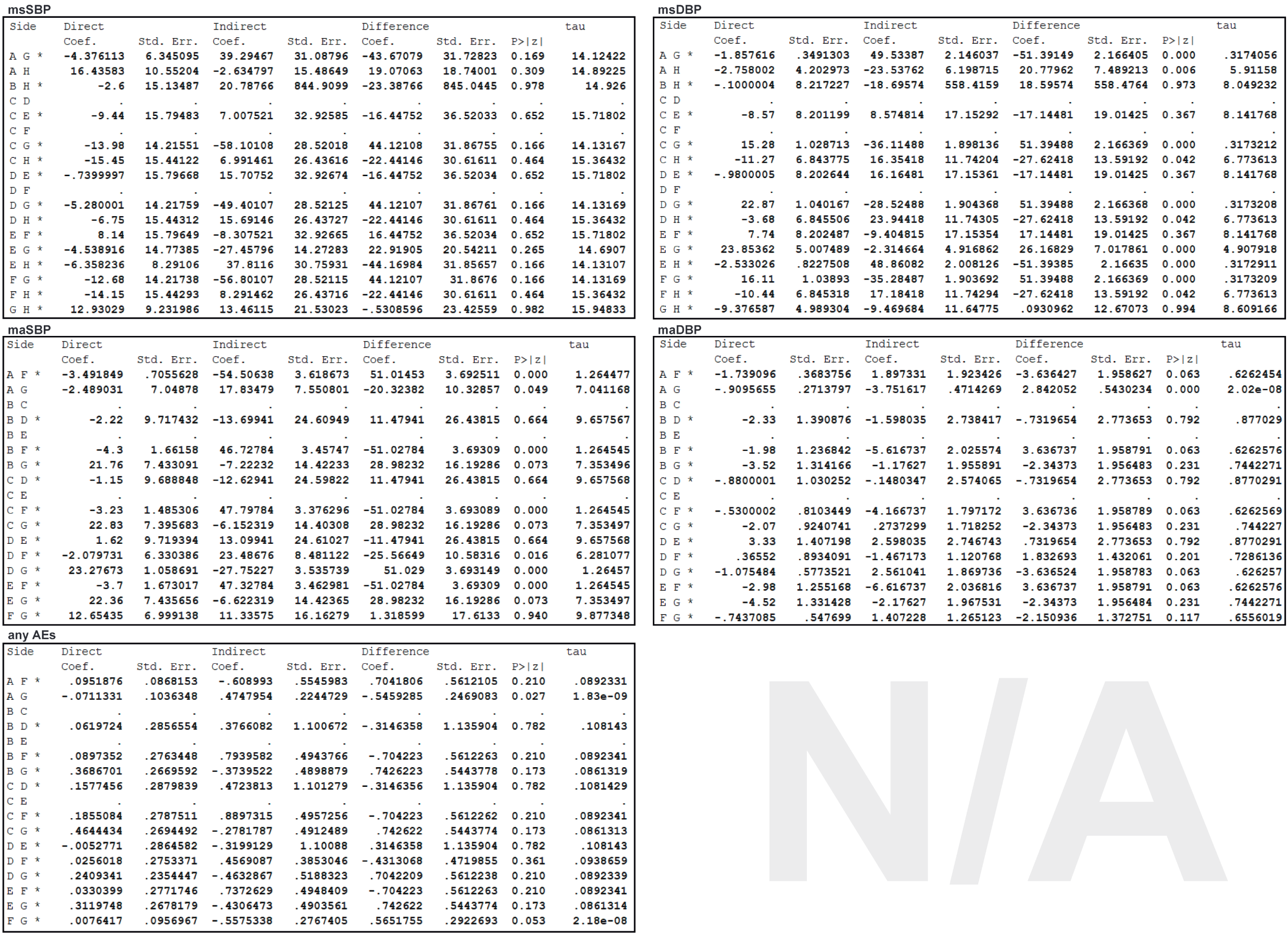


**Figure S3. Local consistency tests for all outcomes.** msSBP, mean systolic blood pressure in the sitting position; msDBP, mean diastolic blood pressure in the sitting position; maSBP, mean ambulatory systolic blood pressure; maDBP, mean ambulatory diastolic blood pressure; AEs, adverse events.


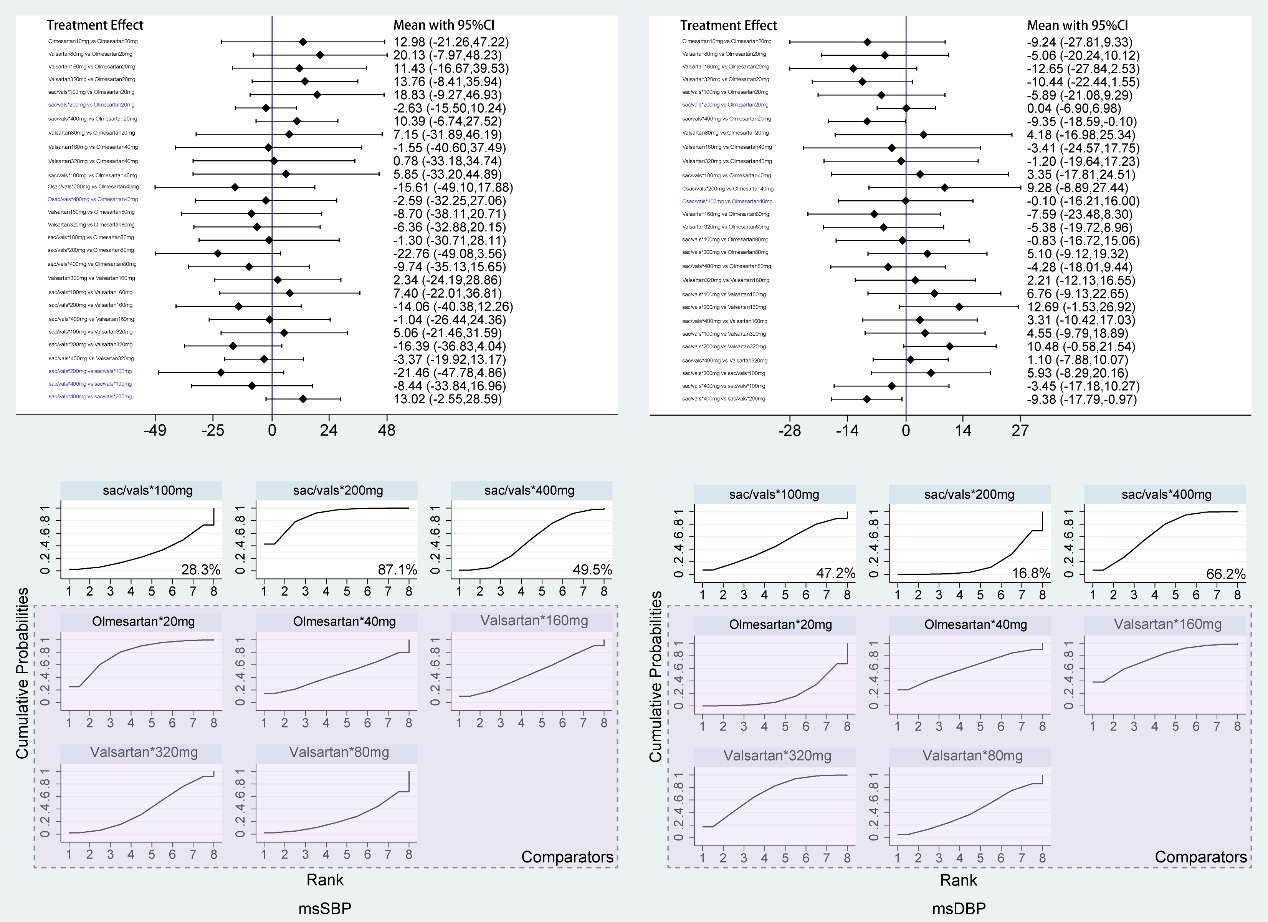


**Figure S4. Forest plots and SUCRA graphes for msSBP and msDBP.** Sac/vals, sacubitril/valsartan; msSBP, mean systolic blood pressure in the sitting position; msDBP, mean diastolic blood pressure in the sitting position.


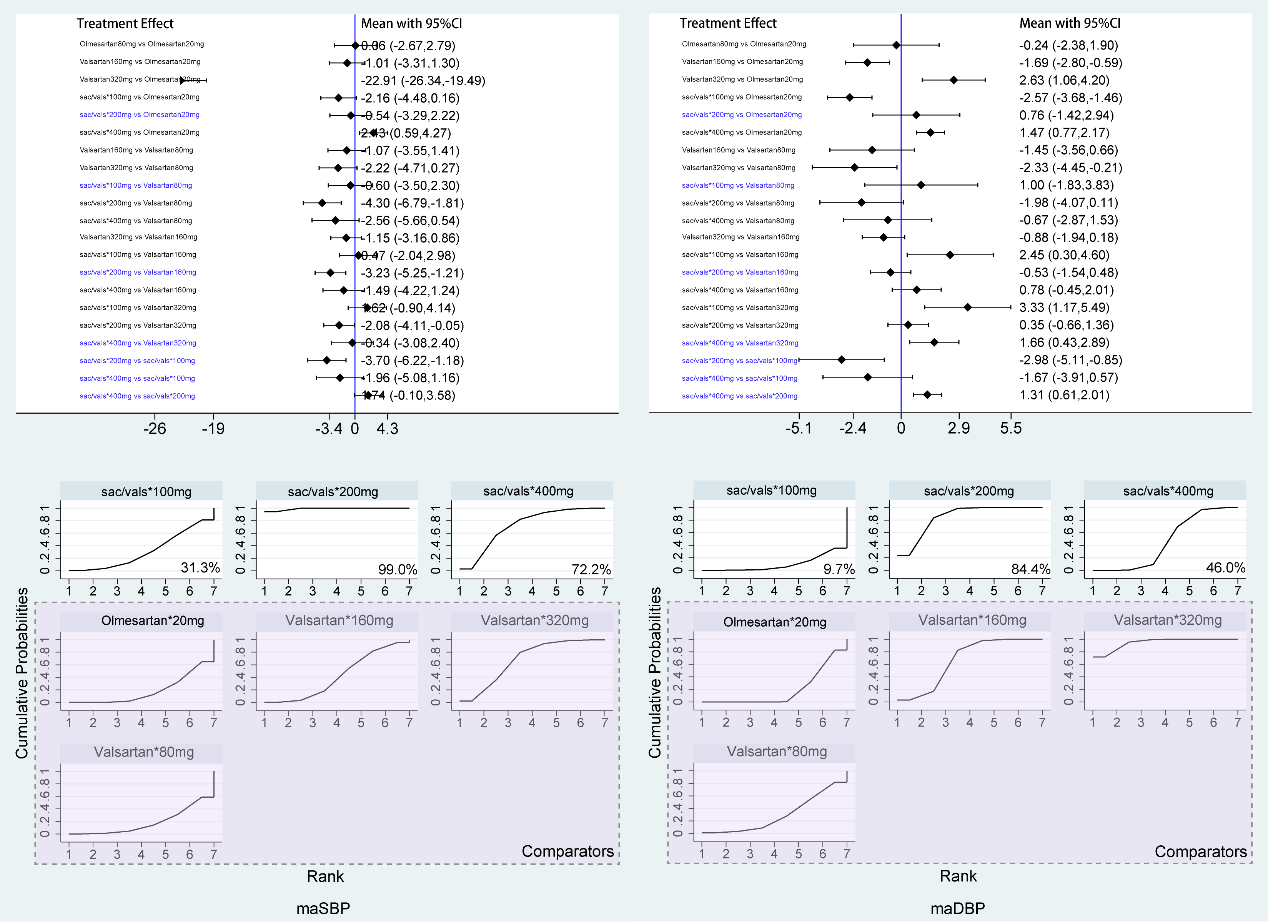


**Figure S5. Forest plots and SUCRA graphes for maSBP and maDBP.** Sac/vals, sacubitril/valsartan; maSBP, mean ambulatory systolic blood pressure; maDBP, mean ambulatory diastolic blood pressure.


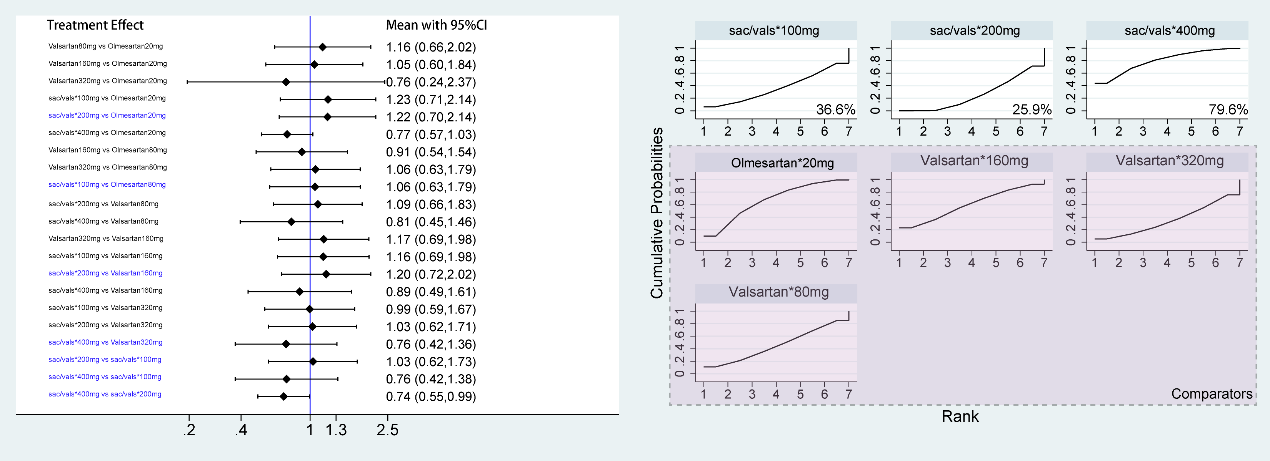


**Figure S6. Forest plots and SUCRA graphes for trial-designed AEs.** Sac/vals, sacubitril/valsartan; AEs, adverse events.


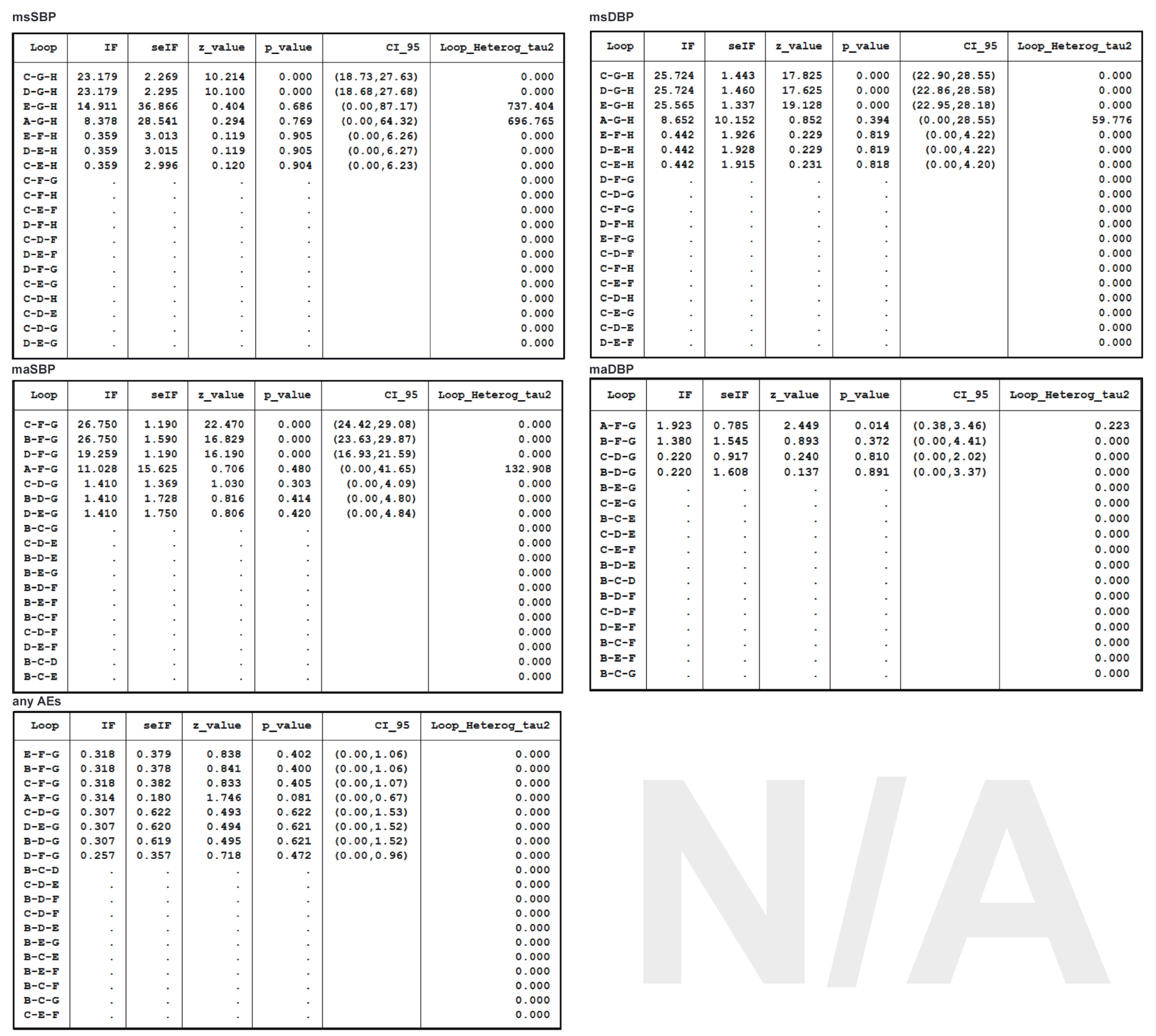


**Figure S7.** **Inconsistency plots for all outcomes**. msSBP, mean systolic blood pressure in the sitting position; msDBP, mean diastolic blood pressure in the sitting position; maSBP, mean ambulatory systolic blood pressure; maDBP, mean ambulatory diastolic blood pressure; AEs, adverse events.


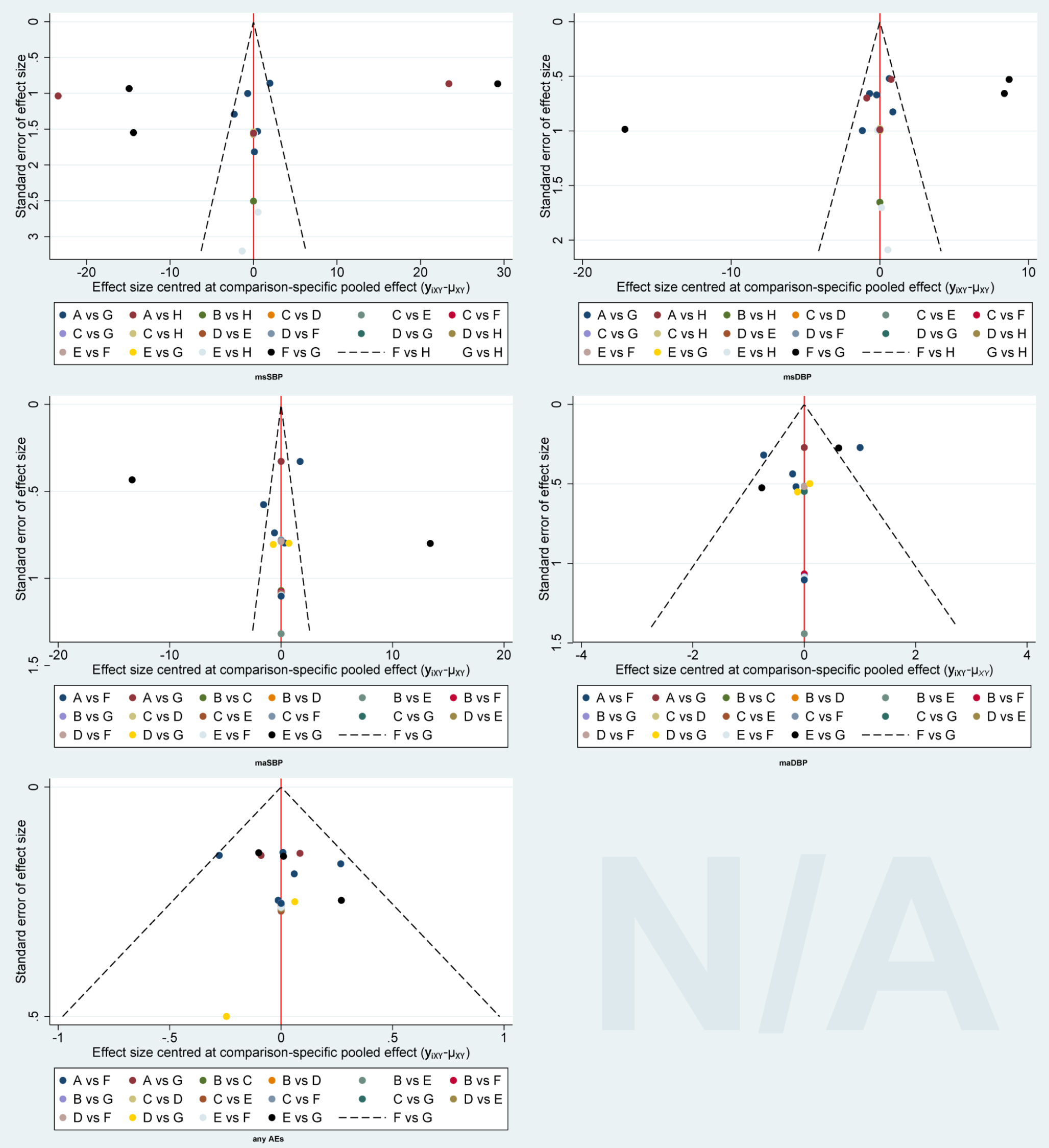


**Figure S8. Comparison-adjusted funnel plot for all outcomes.** msSBP, mean systolic blood pressure in the sitting position; msDBP, mean diastolic blood pressure in the sitting position; maSBP, mean ambulatory systolic blood pressure; maDBP, mean ambulatory diastolic blood pressure; AEs, adverse events.

**Table S1 Detailed search strategy of target databases.**

**Search strategy of PubMed**

| No. | Query | Results |
| --- | --- | --- |
| #8 | ((("sacubitril and valsartan sodium hydrate drug combination" [Supplementary Concept]) OR (((((sacubitril-valsartan[Title/Abstract]) OR (sacubitril/valsartan[Title/Abstract])) OR (Entresto[Title/Abstract])) OR (AHU377[Title/Abstract])) OR ("sacubitril valsartan"[Title/Abstract]))) AND (("Hypertension"[Mesh]) OR ((hypertension[Title/Abstract]) OR (high blood pressure[Title/Abstract])))) AND (random*) | 53 |
| #7 | random* | 1,564,671 |
| #6 | ("Hypertension"[Mesh]) OR ((hypertension[Title/Abstract]) OR (high blood pressure[Title/Abstract])) | 529,375 |
| #5 | (hypertension[Title/Abstract]) OR (high blood pressure[Title/Abstract]) | 442,667 |
| #4 | "Hypertension"[Mesh] | 307,019 |
| #3 | ("sacubitril and valsartan sodium hydrate drug combination" [Supplementary Concept]) OR (((((sacubitril-valsartan[Title/Abstract]) OR (sacubitril/valsartan[Title/Abstract])) OR (Entresto[Title/Abstract])) OR (AHU377[Title/Abstract])) OR ("sacubitril valsartan"[Title/Abstract])) | 1,533 |
| #2 | ((((sacubitril-valsartan[Title/Abstract]) OR (sacubitril/valsartan[Title/Abstract])) OR (Entresto[Title/Abstract])) OR (AHU377[Title/Abstract])) OR ("sacubitril valsartan"[Title/Abstract]) | 1,391 |
| #1 | "sacubitril and valsartan sodium hydrate drug combination" [Supplementary Concept] | 920 |

**Search strategy of Embase**

| NO. | Query | Results |
| --- | --- | --- |
| #9 | #8 AND [embase]/lim | 189 |
| #8 | #3 AND #6 AND #7 | 210 |
| #7 | random* | 2,039,628 |
| #6 | #4 OR #5 | 1,082,266 |
| #5 | 'hypertension'/exp | 867,568 |
| #4 | hypertension:ti,ab,kw OR 'high blood pressure':ti,ab,kw | 700,802 |
| #3 | #1 OR #2 | 3,959 |
| #2 | 'sacubitril plus valsartan'/exp | 3,688 |
| #1 | 'sacubitril valsartan':ti,ab,kw OR 'sacubitril/valsartan':ti,ab,kw OR entresto:ti,ab,kw OR ahu377:ti,ab,kw OR 'sacubitril plus valsartan':ti,ab,kw OR lcz696:ti,ab,kw | 2,706 |

.......................................................

**Search strategy of Cochrane library**

| NO. | Query | Results |
| --- | --- | --- |
| #1 | (sacubitril):ti,ab,kw AND (valsartan):ti,ab,kw | 498 |
| #2 | (sacubitril valsartan):ti,ab,kw OR (Entresto):ti,ab,kw OR (AHU377):ti,ab,kw | 614 |
| #3 | #1 or #2 | 614 |
| #4 | (hypertension):ti,ab,kw OR (high blood pressure):ti,ab,kw | 83489 |
| #5 | MeSH descriptor: [Hypertension] explode all trees | 19722 |
| #6 | #4 or #5 | 83489 |
| #7 | #3 and #6 | 86 |
